# Overview of Recent Advances in Canine Parvovirus Research: Current Status and Future Perspectives

**DOI:** 10.3390/microorganisms13010047

**Published:** 2024-12-30

**Authors:** Hongzhuan Zhou, Kaidi Cui, Xia Su, Huanhuan Zhang, Bing Xiao, Songping Li, Bing Yang

**Affiliations:** 1 Beijing Key Laboratory for Prevention and Control of Infectious Diseases in Livestock and Poultry, Institute of Animal Husbandry and Veterinary Medicine, Beijing Academy of Agriculture and Forestry Sciences, Beijing 100097, China; hanfu2002@sohu.com (H.Z.); ckd2024syphu@163.com (K.C.); sxia2013@163.com (X.S.); zhh20qe@163.com (H.Z.); xiaobing1972@sohu.com (B.X.); 2 School of Life Science and Biopharmaceutics, Shenyang Pharmaceutical University, Shenyang 117004, China

**Keywords:** canine parvovirus, research advances, pathogenesis, antiviral drugs

## Abstract

Canine parvovirus (CPV-2) was first identified in the late 1970s and has since become one of the most significant infectious agents affecting dogs. CPV-2 causes severe diseases such as hemorrhagic gastroenteritis and myocarditis, posing a major threat to canine health, particularly with a high mortality rate in puppies. It is globally recognized as a highly contagious and lethal pathogen. CPV is prone to rapid mutation, leading to the emergence of new variants. Despite widespread vaccination efforts, CPV remains one of the primary causes of acute gastroenteritis and death in young and juvenile dogs. Furthermore, the detection of CPV in swine populations has introduced additional challenges to its control. This review summarizes the current epidemiological status of CPV, highlighting recent advancements in diagnostic techniques and vaccine development. Additionally, it discusses the latest research on the pathogenesis of the virus and the development of antiviral agent research and proposes prevention and control suggestions for CPV under the One Health concept. In particular, there is a need to enhance surveillance of viral dynamics, accelerate the development of novel vaccines, and deepen the exploration of the underlying pathogenic mechanisms. This review aims to provide a scientific foundation for effective control of CPV and to guide future research directions.

## 1. Introduction

Parvoviruses are ancient viruses capable of infecting a wide range of animals, including humans, and targeting various tissue types [1]. In recent years, there has been a notable increase in outbreaks of human parvovirus [2], the discovery of new strains such as porcine parvovirus PPV8, and the emergence of a novel goose parvovirus associated with short beak and short body syndrome [3,4]; the cross-species transmission of canine parvovirus (CPV) (e.g., to Artiodactyla-pigs) has also been reported [5], indicating that the prevention and control situation of parvovirus has become more complex and challenging.

It is particularly important to provide a comprehensive review of parvoviruses in response to new situations. In this article, we focus on the CPV. CPV-2 was first isolated and identified in the late 1970s and has since evolved into multiple new variants of CPV-2a, CPV-2b, CPV-2c, new CPV-2a, and new CPV-2b [6]. CPV can induce hemorrhagic gastroenteritis and myocarditis in dogs, which pose significant health risks to affected animals [7]. The mortality rate in unvaccinated puppies can be as high as 91% [8]. As dogs are an increasingly important companion species, dogs’ health status has attracted more and more attention. Since the CPV virus has the ability to survive in the environment for a long time, it represents a continuous threat to canine health, which will in turn adversely affect the development of dog breeding and pet industries and cause economic losses [9]. This article integrates the latest progress in canine parvovirus research and discusses the epidemic status, biological characteristics, pathogenesis, diagnostic methods, prevention and control, etc., with the aim of providing a scientific basis for the future prevention and control of CPV.

## 2. Biological Properties of Viruses

CPV belongs to the species *Carnivore protoparvovirus 1*, genus *Protoparvovirus*, and family *Parvoviridae* [10]. Based on a comprehensive phylogenetic analysis of CPV, raccoon parvovirus (RPV), feline panleukopenia virus (FPV), mink enteritis virus (MEV), and blue fox parvovirus (BFPV), it was found that these viruses share a common ancestral origin [11].

CPV is a small, non-enveloped virus characterized by an icosahedral capsid with a diameter of approximately 26 nm (T = 1 symmetry). Its genome consists of a linear, single-stranded negative-sense DNA approximately 5 kb in length [12]. The virus encodes two non-structural proteins (NS1 and NS2) and two structural proteins (VP1 and VP2) within two open reading frames [13]. VP1 and VP2 are major structural proteins that form the viral capsid, produced through selective splicing of mRNA. Among them, VP2 accounts for approximately 90% of the viral capsid [14]. These structural proteins are composed of eight anti-parallel β-barrel domains. The β-strands are connected by loops, forming the surface of the capsid. The viral particles exhibit spike-like protrusions around the triplet axis, dimple-like depressions at the doublet axis, and cylindrical channels (canyons) at the fivefold axis [15,16].

CPV-2 evolved from feline panleukopenia virus (FPV), with key amino acid substitutions identified at the following positions: Arg80Lys, Lys93Asn, Val103Ala, Asp323Asn, Asn564Ser, and Ala568Gly [17]. Although CPV is a DNA virus, CPV exhibits a mutation rate in amino acid sequences similar to that of RNA viruses [18]. Compared with the original strain CPV-2, CPV-2a has mutations at five amino acid positions: Met87Leu, Ile101Thr, Ala300Gly, Asp305Tyr, and Asn375Asp. CPV-2a (426Asn), CPV-2b (426Asp), and CPV-2c (426Glu) are distinguished by mutations at residue 426 [19]. The Ser297Ala mutation is considered to be a sign of new CPV-2a/2b [20]. In addition to the above mutation sites used to distinguish antigen typing, amino acid mutations in the VP2 protein will also occur at sites 5, 267, 324, 370, 440, and 481 [21,22]. Specifically, Ala5Gly, Thr440Ala, and Arg481Lys may alter the antigenic structure and immunogenicity of the virus. Y324I and Gln370Arg may affect the host range of viral infection by affecting binding to the receptor [7,23].

In addition to its natural host, the domestic dog, CPV has been detected in a variety of wild animals, including wolves, badgers, raccoons, otters, pangolins, tigers, foxes, and pandas. The expansion of the CPV host range is primarily attributed to amino acid mutations in the viral capsid protein VP2. Notably, the CPV-2c variant plays a critical role in the virus’s transmission from carnivores to even-toed ungulates and pangolins. Studies have reported that the sequence homology of the human transferrin receptor (TfR) with that of pangolins and pigs is 79.0% and 77.2%, respectively. Furthermore, the amino acid residues involved in virus binding are highly conserved. This suggests that the strains isolated from pangolins and pigs may have the potential to infect humans [24].

## 3. Epidemiological Characteristics and Strain Distribution

CPV primarily exhibits a sporadic pattern of occurrence in natural environments, with both single and mixed infections being commonly observed [25]. However, in areas with high pet trade activity or dense breeding populations, outbreaks may occur [26]. The infection activity of this virus has been recorded throughout the year, and its epidemic intensity is particularly prominent in spring [27]. CPV has a wide range of infectivity of dogs of different ages, genders, and breeds, among which puppies aged 2 to 4 months are a high-risk group for the virus infection. This age group not only experiences higher morbidity but also faces an increased mortality rate. In terms of gender differences, male dogs are more susceptible to infection than female dogs. In addition, native dogs and mixed-breed dogs tend to have stronger resistance to CPV compared to purebred dogs [28].

CPV undergoes continuous genetic variation and natural selection, exhibiting a high degree of evolutionary adaptability. In different regions around the world, multiple antigenic variant strains are widely circulated with different combination patterns and distribution frequencies. As of November 2024, we have retrieved complete genome sequences from 261 strains of CPV, representing 9 different species and 23 countries, from the GenBank database. Subsequently, we conducted supplementary identification of variant classifications for 132 of the strains based on specific mutation sites on the amino acid sequence of the VP2 protein (Supplemental Table S1) [29]. Statistical analysis revealed that the majority of the CPV sequences obtained were from China, followed by Uruguay, the United States, Peru, Canada, and Brazil. The highest number of viral sequences was provided by Asia (99 sequences), followed by South America (91 sequences), North America (44 sequences), Africa (8 sequences), Europe (7 sequences), and Oceania (4 sequences). Further analysis of the composition of the 261 complete CPV genome sequences revealed that the proportions of CPV-2a (37.55%) and CPV-2c (36.02%) were similar, both significantly higher than those of CPV-2b (14.17%) and CPV-2/2-like (12.26%). CPV-2a is the predominant variant in Asia, whereas CPV-2c is the most prevalent variant in South America. Although CPV-2a remains the globally dominant strain, there is a noticeable trend of CPV-2c gradually replacing CPV-2a as the predominant variant in certain regions.

**Figure 1:**
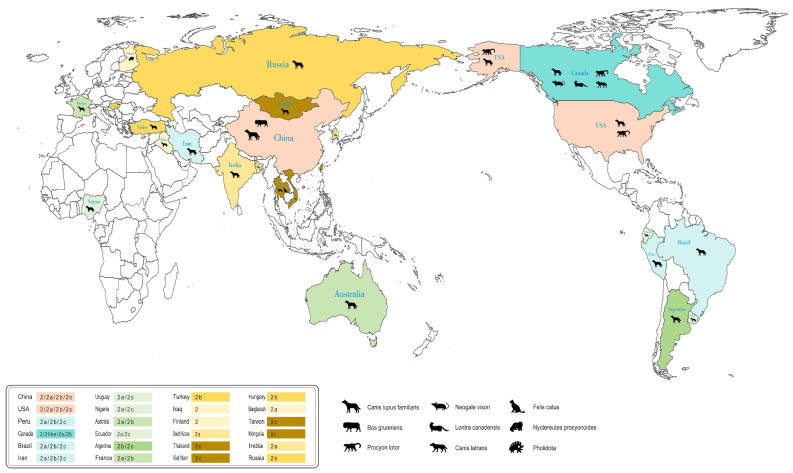
Global distribution of canine parvovirus. The complete genome sequences of Canine Parvovirus (CPV) available in GenBank were analyzed using SnapGene (Version 5.1.5) and WPS Office (Version 12.1.0.16417). Different colors are used to represent various genotype types, with the intensity of the color reflecting the different genotype combinations within the same type. Different species are represented by corresponding animal images.

While analyzing the global distribution of CPV variants, we also conducted a comprehensive examination of host distribution (Figure 1). Notably, Canada exhibited the greatest diversity of host species, covering not only common hosts such as dogs, but also a variety of wild animals such as procyon lotor, neogale vison, lontra canadensis, and canis latrans. This highlights the need for enhanced surveillance of CPV in wildlife populations within Canada. Furthermore, our findings indicate the presence of canine parvovirus in other species beyond those mentioned above, including bos grunniens, felis catus, nyctereutes procyonoides, and pholidota, which further underscores the wide host range of the virus.

## 4. Pathogenesis of CPV

The most common route of transmission for CPV is fecal-oral contact or exposure to contaminated surfaces. Following infection with CPV, the virus predominantly targets rapidly dividing cells, primarily intestinal epithelial cells and lymphocytes. In young puppies, myocardial cells are also in a phase of active division, making them susceptible to CPV infection. This contributes to the development of myocarditis in infected puppies, which is one of the key factors underlying the high mortality rate observed in this age group [30,31]. Viral multiplication in rapidly dividing intestinal epithelial cells leads to intestinal hyperdynamism, inflammation, and tissue necrosis, which in turn may cause hemorrhagic enteritis. This process can trigger systemic inflammatory response syndrome (SIRS), septicemia, and endotoxemia. Furthermore, viral replication in the bone marrow and lymphoid tissues can lead to leukopenia, thereby further impairing the host’s immune function [11,32].

The interaction between viruses and their hosts is primarily manifested through the viral proteins’ manipulation of host cellular molecular mechanisms. The non-structural protein NS1 plays a critical role in this process. NS1 exploits the host cell’s replication and transcription machinery, utilizing two DNA-binding domains to regulate the replication of the viral genome and the activation of the P38 promoter [33]. Furthermore, NS1 induces a shutdown of host translation by attenuating mTOR phosphorylation, thereby impairing the host immune response and contributing to the pathogenic effects [34].

Early mechanism research on CPV mainly focused on the impact of key residues of structural proteins on virus proliferation and transmission. For example, basic region 1 (BR1) in the VP1 capsid protein has a nuclear localization signal (NLS) function, and the 300th amino acid in VP2 determines host specificity [35,36]. Additionally, studies have reported that CPV virus particles can specifically bind to the transferrin receptor (TfR) on the surface of host cells, thereby triggering the clathrin-mediated endocytosis process. The viral particles are internalized in endosomes and transported to the lysosome via this pathway. Following this, they are released from the lysosome into the cytoplasm and eventually transported to the cell nucleus [37]. Recently, studies have reported that when the virus binds to TfR, the epidermal growth factor receptor (EGFR) that interacts with TfR will undergo changes in the tyrosine phosphorylation site, leading to G1/S cell cycle arrest of the host cell. This in turn mediates the replication of CPV [38].

The invasion of CPV can activate the host cell’s apoptotic pathways, including intrinsic and extrinsic pathways. It involves the activation of caspase-3, caspase-8, caspase-9, and caspase-12, a decrease in mitochondrial potential, and an increase in the Bax/Bcl2 ratio [39]. Studies have shown that the overexpression of NS1 is closely related to the arrest of the cell cycle at the G1 phase, leading to mitochondrial depolarization, the release of cytochrome C, activation of caspase-9, and the accumulation of reactive oxygen species (ROS) levels [40]. In addition, NS1 can cause HeLa cell apoptosis, and this process does not depend on the p53 pathway [41].

**Figure 2:**
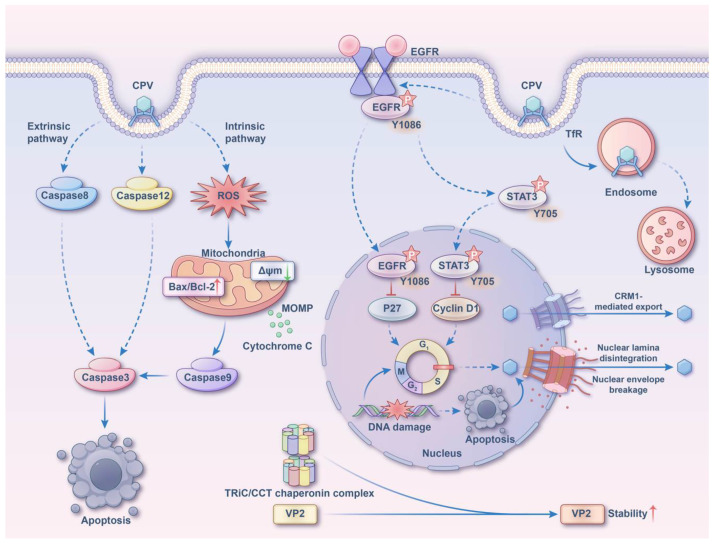
CPV-infection-mediated apoptosis and virus-host-cell interactions. (i) CPV infection activates caspase-8 and caspase-12, leading to the accumulation of reactive oxygen species (ROS). This accumulation results in a decrease in mitochondrial membrane potential, which triggers the release of cytochrome c and activates caspase-9. Ultimately, both caspase-8 and caspase-9 collaboratively upregulate the expression of caspase-3, thereby inducing apoptosis. (ii) CPV binds to the TfR receptor, which facilitates its internalization via endocytic transport to the lysosome. Additionally, this interaction activates the EGFR (Y1086)/p27 and STAT3 (Y705)/cyclin D1 signaling axes, leading to cell cycle arrest. (iii) CPV entry into host cells induces DNA damage, which subsequently triggers cell cycle arrest and apoptosis. This process leads to an increase in nuclear membrane permeability, allowing the viral capsid to escape via a CRM1-independent nuclear export pathway. (iv) The CPV coat protein VP2 is able to increase its own stability by binding to CCT7. The chart is based on content or images compiled from the cited articles [42,43,44,45,46].

In addition to the above studies, Mattola S et al. (2022) reported that after CPV completes replication and assembly in the cell nucleus, the nuclear escape of the CPV capsid depends not only on the CRM1-mediated nuclear export pathway but also on the activation of the G2/M checkpoint and the increased nuclear membrane permeability induced by apoptosis, which facilitates the passive diffusion of the capsid [42]. Furthermore, Mattola S et al. (2022) also revealed the potential role of NS2 in chromatin remodeling and DNA damage response [43]. The study by Su et al. (2024) reported that the chaperonin TRiC/CCT subunit CCT7 can interact with the VP2 protein, enhancing its stability and thereby promoting the replication of CPV (Figure 2) [44].

Overall, research on the molecular pathogenic mechanisms related to CPV remains limited. Due to the lack of antibodies against specific proteins or protein modifications in canine and cat host cells, it is difficult to experimentally verify the functions of some specific proteins or the roles of related pathways. This, to some extent, limits further research on the pathogenesis of CPV.

## 5. Detection of CPV

The presumptive diagnosis of CPV infection is typically based on clinical signs and physical examination. In the context of the complex pathophysiology of CPV infection, identifying suitable biomarkers plays a crucial role in guiding appropriate therapeutic strategies and improving the survival rates of affected dogs. The study found that serum CK-MB concentration, salivary choline, and absolute neutrophil count were the most useful biological biomarkers for determining the severity of CPV infection [32]. However, presumptive diagnosis may not be reliable in all cases. Therefore, a definitive diagnosis still requires the integration of virological, immunological, and molecular biological methods.

Virus isolation and culture technology remain the gold standard for CPV diagnosis. Traditional diagnostic methods include immunological detection techniques, such as latex agglutination test, hemagglutination and hemagglutination inhibition tests, enzyme-linked immunosorbent assay (ELISA), etc., as well as molecular biology techniques, including PCR, Real-time PCR, multiplex PCR (mPCR), loop-mediated isothermal amplification (LAMP), polymerase spiral reaction (PSR), recombinase polymerase amplification assay (RPA), etc. [11]. Among them, PCR is known for its high sensitivity and specificity, making it an effective tool for detecting various strains of CPV-2 and its variants. Therefore, researchers have developed a variety of single-pass or multi-pass detection methods based on PCR. Dema et al. (2021) reported an ARMS-PCR single-step detection method that can quickly and efficiently distinguish CPV-2 antigen types without relying on sequencing [47]. Thieulent et al. (2023) developed a one-step multiplex RT-qPCR assay kit for the simultaneous detection of six canine enteric viruses, including CPV, four feline enteric viruses including FPV, and SARS-CoV-2 virus [45]. In clinical diagnostics, test kits based on enzyme-linked immunosorbent assay (ELISA) and test strips developed using colloidal gold immunochromatographic technology are commonly employed. Compared to ELISA, colloidal gold test strips offer a more rapid, cost-effective, and efficient method for detecting canine parvovirus (CPV), particularly in field settings [48,49]. Furthermore, researchers have not only developed highly sensitive and specific colloidal gold immunochromatographic strips (ICS) but also introduced a colloidal gold-based competitive lateral flow assay (cLFA) system [49,50].

In order to develop more convenient, rapid, and efficient CPV detection methods, researchers have conducted several exploratory studies in recent years: (i) Chen et al. (2024) established one-tube LAMP-CRISPR/Cas12b nucleic acid extraction and detection platform based on magnetic nanoparticle enrichment technology. This method achieves a detection limit as low as 10^−1^ copies/μL, with sensitivity 100 times greater than that of qPCR and LAMP [51]. (ii) The Accelerated Denaturation Bubble-Mediated Strand Exchange Amplification (ASEA) technique developed by Hou et al. (2020) enables rapid detection of the CPV genomic DNA from mixtures within 20 min. This method demonstrates higher sensitivity compared to colloidal gold-based lateral flow assays [52]. (iii) Xu et al. (2020) proposed an improved polymerase cross-linking spiral reaction (PCLSR) method. This method requires only a 50 min reaction at 62 °C in a water bath, and the results can be directly observed with the naked eye. Its accuracy is superior to that of colloidal gold detection [53]. (iv) The CRISPR-Cas13a-mediated nanosystem designed by Khan H et al. (2019) can detect 100 amol/L CPV-2 DNA within 30 min without complex instruments [54]. These studies provide more options to fulfill the detection needs in different application scenarios.

## 6. Prevention and Control

### 6.1. Treatment

#### 6.1.1. Supportive Treatment

CPV infection is widespread globally, and current treatment methods are primarily based on symptomatic supportive therapy. Vomiting and acute hemorrhagic gastroenteritis are the main clinical symptoms of CPV infection. CPV infection can damage gastrointestinal epithelial cells, leading to bacterial translocation, which in turn causes sepsis and endotoxemia. Consequently, fluid therapy, effective antiemetics, and antibiotics are essential components in the management of CPV infection [46]. The standard clinical treatment protocol is typically as follows: intravenous administration of crystalloids (such as Ringer’s lactate), supplemented with potassium chloride, is provided based on individual needs. For dogs with severe hypovolemia or hypoproteinemia, synthetic colloids are used. Enteral feeding is temporarily withheld for 1–2 days, while antiemetic drugs (e.g., metoclopramide, administered every 6–8 h) and antibiotics (e.g., enrofloxacin, administered once every 24 h) are administered [55,56]. To enhance the therapeutic efficacy, complementary therapies are often employed, including ozone therapy, the use of immunomodulators, the administration of probiotics, and the supplementation of antioxidants [57,58,59,60]. However, it is important to note that supportive therapy is primarily aimed at symptom relief and does not offer a specific antiviral treatment.

#### 6.1.2. Antiviral Agents

**Figure 3:**
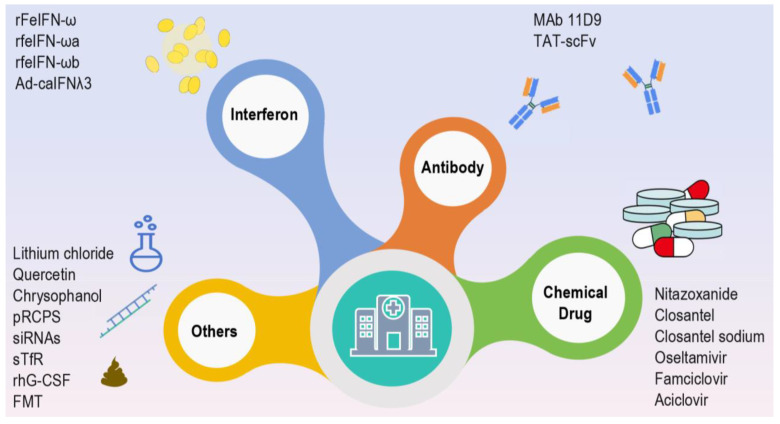
Potential antiviral agents against CPV. The current research on antiviral agents is primarily focused on four main categories: interferon-based formulations, antibody-based therapies, chemical drugs, and other types of treatments. The insights are visually represented via the “iSlide” plugin downloaded from https://www.islide.cc (accessed on 20 November 2024).

Although the CPV vaccine has been recognized as a core vaccine by international professional organizations such as the World Small Animal Veterinary Association (WSAVA) and American Animal Hospital Association (AAHA) and widely used in the vaccination of dogs, CPV remains a major cause of high mortality rates in juvenile dogs, even in developed countries with high vaccination coverage [61]. Currently, the treatment of CPV mainly relies on symptom-based supportive therapy; however, in individuals with higher viral loads, existing therapeutic interventions often fail to effectively control the disease. Therefore, an increasing number of researchers are focusing on the development of antiviral agents, laying a solid foundation for the optimization of future treatment strategies for CPV infection. Through a systematic review, current research on antiviral agents can be mainly classified into the following categories. (i) Research on interferon-based formulations, such as recombinant feline interferon-omega (which improved survival rates in treated dogs from 57.1% to 85.7% compared to the control group), feIFN-ωa and feIFN-ωb, and Ad-caIFNλ3, etc. [46,62,63]. (ii) Research on antibody-based therapies, such as canine-derived chimeric MAb 11D9 and TAT-scFv (transactivating transcriptional activator-scFv), etc [64,65]. (iii) Research on chemical drugs, such as nitazoxanide, closantel sodium, closantel (the 50% antiviral efficacy concentration (EC50) values for nitazoxanide, closantel sodium, and closantel in inhibiting CPV in vitro were 2.71 µM, 6.01 µM, and 7.77 µM, respectively [66]), oseltamivir (which increased survival rates from 57.1% to 71.4% in treated dogs compared to controls), famciclovir (which also raised survival rates from 57.1% to 71.4%), acyclovir, etc. [11,67,68]. (iv) Other relevant studies such as compounds or active extracts (lithium chloride, quercetin, chrysophanol, phosphorylated Radix Cyathulae officinalis polysaccharides (pRCPS), etc.), small interfering RNAs, soluble canine transferrin receptor, recombinant human granulocyte colony-stimulating factor, and fecal microbiota transplantation (which reduced mortality from 36.4% to 21.2% in treated dogs compared to the control group), etc. [67,69,70,71,72]. (Figure 3). Although some antiviral agents have undergone clinical trials, the majority remain at the in vitro testing stage. Therefore, the potential for their clinical application requires further validation through clinical studies. Additionally, the efficacy of antiviral drugs in the treatment of severe cases remains to be fully assessed.

### 6.2. Vaccines

Currently, the most effective way to prevent CPV infection remains vaccination. CPV vaccines are widely recommended as one of the core vaccines for pet dogs, and they play an important role in preventing CPV-associated diseases [73]. Vaccination strategies should take individual factors into account, including body weight, age, nutritional status, and the prevalence of canine parvovirus (CPV) in different regions. As such, there is no universal vaccination protocol that can address all circumstances [74]. Antibody testing should be conducted prior to vaccination. On one hand, testing for maternally derived antibodies can help determine the optimal timing for the first vaccination in puppies, thereby minimizing the interference of maternal antibodies on vaccine efficacy [75]. On the other hand, considering that aging and the passage of time post-vaccination can impact immune responses, measuring protective antibody titers can aid in adjusting the vaccination intervals for adult dogs [76,77]. Additionally, studies have shown that CPV incidence is higher in regions with poorer socio-economic conditions, where inadequate vaccination coverage may contribute to insufficient herd immunity, thereby exacerbating the prevalence of the disease [78]. It is also important to note that new CPV variants continually emerge, exhibiting changes in antigenicity, immunogenicity, and virulence, which may challenge the effectiveness of existing vaccines and potentially lead to vaccination failures [17]. Therefore, regular molecular surveillance of circulating CPV strains, pre-vaccination antibody testing, and the implementation of widespread vaccination programs in high-risk areas will be essential for achieving herd immunity and effectively preventing the spread of CPV.

It is worth noting that, despite the implementation of intensive vaccination programs in developed countries, CPV infections still occur from time to time [61]. Studies have shown that interference from maternally derived antibodies (MDAs) may be one of the key factors affecting vaccine efficacy or leading to vaccine failure in puppies [79]. Therefore, researchers explore the use of new technologies to address this issue. Pearce J et al. (2023) developed a novel recombinant canine parvovirus type 2c vaccine strain. This vaccine can overcome the interference of maternal antibodies and stimulate protective immunity in four-week-old puppies [80]. In addition, with the continuous emergence of multiple variants of CPV, the protective effect of traditional vaccines against variants is also facing challenges. Therefore, novel CPV vaccines based on virus-like particles (VLPs) with high immunogenicity and safety, as well as multi-epitope vaccines designed using computational methods and immunoinformatics to target highly conserved capsid protein (VP2) regions, could serve as potential candidate vaccines for the prevention of CPV [81,82,83,84].

### 6.3. Prevention and Control Strategies in the Context of “One Health”

**Figure 4:**
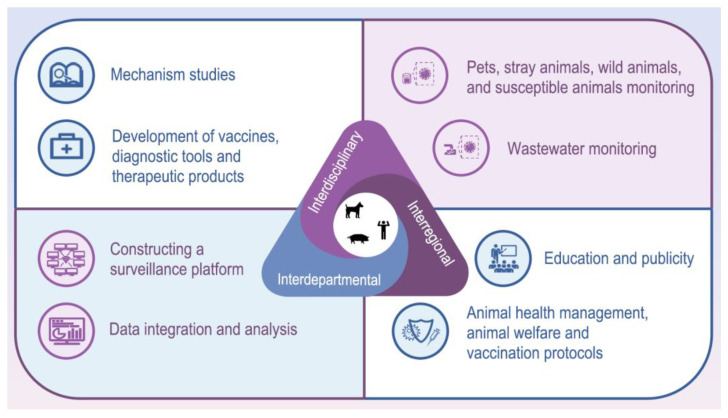
Comprehensive prevention and control of CPV under the concept of “One Health”. By fostering interdisciplinary, intersectoral, and interdepartmental collaborations, new perspectives and strategies are offered for the effective management of CPV. The insights are visually represented using the “iSlide” plugin downloaded from https://www.islide.cc (accessed on 20 November 2024).

CPV has a broad host range, with recent reports indicating its spillover into pigs within the order Artiodactyla [5]. Further research showed that the key amino acid sites of the virus receptor in pigs and humans are highly homologous, posing a potential risk of cross-species transmission to humans [24]. The interspecies transmission of canine parvovirus (CPV) in susceptible animal hosts poses a potential threat to both animal and human health in the future. Therefore, the establishment of a comprehensive monitoring system is of paramount importance, one that can promptly detect and respond to emerging risks, thereby effectively safeguarding the health of both animals and humans. “One Health” is an interdisciplinary approach that involves collaboration across veterinary science, public health, social sciences, information science, and artificial intelligence, as well as coordination across sectors such as agricultural health and research education. The goal of this approach is to optimize overall health by integrating the well-being of humans, animals, and ecosystems. This framework is critical for controlling the spread of CPV within both domestic and wild animal populations. The measures that can be taken are as follows: (i) Research and education departments should strengthen studies on the pathogenic mechanisms of CPV, particularly the mechanisms of cross-species transmission, and develop improved vaccines, diagnostic tools, and therapeutic products. (ii) Construct a comprehensive CPV surveillance network platform. Utilizing information technology to integrate data from multiple departments such as animal healthcare, research, and disease control across various regions. Monitor and analyze the virus’s characteristics in different seasons, geographic areas, and susceptible species, providing a scientific basis for early warning and outbreak prediction. (iii) Strengthen the monitoring of pets, stray animals, wild animals, susceptible animals, and wastewater around the breeding environment. (iv) Health management departments should strengthen the formulation and publicity guidance of policies on the health management of dogs and cats, animal welfare, vaccination protocols, etc. (Figure 4).

## 7. Future Outlook

**Figure 5:**
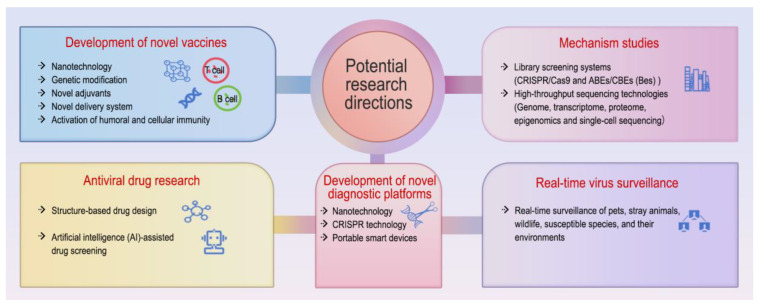
Potential future research directions for CPV. (i) Development of novel vaccines. (ii) Studies on the evolution, transmission, and pathogenesis of CPV. (iii) Research on antiviral drug development. (iv) Construction of new diagnostic platforms. (v) Real-time monitoring of viral infections. The insights are visually represented using the “iSlide” plugin downloaded from https://www.islide.cc (accessed on 20 November 2024).

It can be seen from existing CPV reports and research that the prevention and control of CPV still face several issues that require in-depth exploration. Addressing these challenges will necessitate closer interdisciplinary collaboration among virologists, immunologists, veterinarians, and public health professionals. Potential future research directions are outlined as follows: (i) Development of novel vaccines. The prevention of CPV mainly relies on vaccination, but due to variants and maternal antibodies, immune failure occurs from time to time [28]. Therefore, one of the important future directions is the development of new vaccines that can effectively activate both humoral and cellular immunity. This could be achieved by utilizing new technologies such as nanotechnology and genetic modification, and the integration of novel adjuvants and delivery systems (for instance, mRNA vaccines, which are both safe and capable of inducing comprehensive antibody responses as well as cellular immunity, represent a promising approach in this regard). (ii) Research on the evolution, transmission, and pathogenic mechanisms of CPV. Further explore the genetic variation and evolutionary mechanisms of CPV-2. Using tools like CRISPR/Cas9 and ABEs/CBEs (Bes) library screening systems to identify host receptors and key amino acid sites of key host receptors during viral infection. Moreover, it is essential to explore the dynamic interactions between the virus (especially the capsid protein) and host receptors, examining their correlation with viral infection, cross-species transmission, and immune evasion. Utilize high-throughput sequencing technologies such as genome, transcriptome, proteome, epigenomic, and single-cell sequencing to explore the interaction between the virus and the host, deeply analyze the host’s immune response to the virus, and identify key viral proteins, pathways, and biological processes involved in immune evasion, and successfully exploit host cellular machinery for proliferation. (iii) Antiviral drug research. As a complement to vaccine-based immunity, it is crucial to employ advanced technologies such as structure-based drug design and artificial intelligence (AI)-assisted drug screening to identify antiviral natural products, bioactive compounds, or drugs that “target the virus” and “target the host”. Develop a series of drugs targeting different stages of the viral life cycle (adsorption, entry, uncoating, replication, assembly, and release) and further explore the antiviral mechanisms and targets of these drugs to provide a theoretical basis for clinical application. In addition, the establishment of a canine myocarditis cell-based drug screening model is recommended to enhance research on canine myocarditis and facilitate the development of novel therapeutic agents. (iv) Development of novel diagnostic platforms. Combining nanotechnology and CRISPR technology to develop rapid, accurate, sensitive, specific, and cost-effective diagnostic methods. Additionally, portable smart devices for data collection should be integrated, facilitating the construction of a detection platform suitable for on-site rapid detection. Improving detection platforms for field-based rapid diagnostics can significantly enhance the capacity for result collection and data analysis, thereby making field surveillance more accurate and efficient. (v) Real-time virus surveillance. Given the bidirectional transmission characteristics of CPV between domestic dogs and wild animals [61], as well as its correlation of detection rates between wild dogs and domestic dogs [85], it is crucial to strengthen real-time surveillance of pets, stray animals, wildlife, susceptible species, and their environments. This surveillance, combined with data from online monitoring platforms, will provide a scientific basis for the epidemiological trends and potential spillover risk assessment of CPV (Figure 5).

## 8. Conclusions

Studies have shown that dog vaccination significantly reduces the infection rate of dogs [86]. However, recent reports of CPV detection in pig herds [5], coupled with the potential risk of human infection [24], indicate that the prevention and control of CPV still face huge challenges. This review provides a brief overview of the current status of the CPV epidemic and introduces new attempts by researchers in detection techniques and vaccine development. At the same time, this article outlines the latest advancements in the study of pathogenic mechanisms and the development of antiviral agents. Then, we provide suggestions for the prevention and control of CPV, as well as suggest potential directions for future research. It is believed that with the continuous progress and innovation of science and technology, we will be able to successfully address the many challenges faced in the prevention and control of CPV.

## Data Availability

The original contributions presented in this study are included in the article/Supplementary Materials. Further inquiries can be directed to the corresponding authors.

## Supplementary Materials

The following supporting information can be downloaded at: https://www.mdpi.com/article/10.3390/microorganisms13010047/s1, Table S1: The complete genome sequences of 261 strains of CPV.
